# Biomarkers in Inflammatory Childhood Diseases

**DOI:** 10.1155/2013/745493

**Published:** 2013-06-10

**Authors:** Wilco de Jager, Dirk Holzinger, Ger T. Rijkers

**Affiliations:** ^1^Centre of Molecular and Cellular Intervention, Department of Paediatric Immunology, University Medical Centre Utrecht, Lundlaan 6, 3584 EA Utrecht, The Netherlands; ^2^Department of Paediatric Rheumatology and Immunology, University Children's Hospital Muenster, Muenster, Germany; ^3^Institute of Immunology, University Hospital Muenster, 48147 Muenster, Germany; ^4^Department of Science, University College Roosevelt, Utrecht University Lange Noordstraat 1, 4330 AB Middelburg, The Netherlands; ^5^Laboratory of Medical Microbiology and Immunology, St. Antonius Hospital, P.O. Box 2500, 3430 EM Nieuwegein, The Netherlands

Biomarkers are of crucial importance in modern medicine for risk-assessment, disease prevention, early diagnosis, drug target identification, drug response and monitoring of disease activity. Biomarkers can be defined as characteristics which are measured and evaluated as indicators of a normal biologic, or pathologic process, or pharmacologic responses towards a therapeutic intervention [1]. Although the term biomarker is relatively new, biomarkers have been used for considerable time, in fact as long as clinical medicine. For example, body temperature is a well-known biomarker for fever; serum levels of C-reactive protein (the most performed assay in hospital laboratories) is a marker for inflammation and glucose levels are used for diagnosis as well as therapeutic readout for controlling diabetes.

For this special issue on Biomarkers in Inflammatory Childhood Diseases we took particular interest in studies dealing with biomarkers for immune mediated childhood diseases. Besides the fact that biomarkers for childhood diseases are, due to ethical restrictions, small population sizes, and limited sample volumes less available, in most cases the normal reference range is extrapolated from adult populations or animal models and therefore not always applicable for paediatric patients. As the pediatric population is in the process of development, both mentally and physically they are at risk for other hazards in comparison with adults. Furthermore, when studying the various parameters of the human immune system changes are observed during aging which have to be taken in account in paediatric patients [2]. Therefore, insight and progression of complex paediatric diseases, in particular inflammatory diseases, can benefit from establishment and validation of a comprehensive set of biomarkers.

In this special Issue on Biomarkers in Inflammatory Childhood Diseases, various paediatric diseases are discussed which are either chronic such as cystic fibrosis and allergic rhinitis or asthma, or acute disease with life threatening complications such as paediatric pulmonary hypertension and H1N1 infection. Of interest to note is that there are several contributions which use or discuss non invasive sample to acquire biological material of a diseased organ, such as exhaled breath condensate from asthmatic children, and faeces of cystic fibrosis patients with potential intestinal inflammation. Furthermore, for optimal data mining several researchers also implemented state of the art new multiplex technologies which can handle micro volumes of biological samples, and produce more insight information in comparison with classical methodologies.

Duncan and colleagues from Colorado, USA, report promising new data on circulating cytokines, in particular EGF, IL-6 and VEGF, which could predict adverse events in children with pulmonary hypertension. In a small cohort of children with H1N1 infection, Chiaretti et al. from Rome, Italy, show that elevated IL-1*β* and IL-6 are associated with disease severity, but not with ultimate outcome. Moed et al. have investigated the value of a set of both cellular as well as cytokine biomarkers in children with allergic rhinitis. While the biomarker profile was of use in establishing the severity of the allergic rhinitis, long term follow-up demonstrated reduction of allergic symptoms with persistence of the in vitro cellular abnormalities. Also in predicting which children with wheezing symptoms would progress to asthma, as was studied by Klaassen et al. in Maastricht, clinical symptoms are better predictors than biomarkers in exhaled breath condensate. In cystic fibrosis, pulmonary inflammation is a major clinical expression of the disease and in the majority of cases respiratory failure is the cause of mortality. Accumulating data now show that intestinal inflammation contributes to the disease and Lee et al. from Sidney, Australia review this evidence, in particular the value of faecal inflammatory markers such as calprotectin and S100A12. The final paper deals with childhood obesity. Adipose tissue is a major source for adipokines, which include hormones such as leptin and adiponectin, cytokines, as well as proteins like apelin and resistin. Machura et al. from Zabrze in Poland have evaluated these adipokines in children with atopic dermatitis and find apelin and visfatin excellent biomarkers for atopic dermatitis.

This special issue on Biomarkers in Inflammatory Childhood Diseases thus reflects the diversity of research in this field and shows the potential of biomarker research in paediatrics. Taken together, the strength of combining non-invasive sampling of biological samples such as saliva, urine, faeces and exhaled breath condensates, as well as multiplex analysis methodologies which can handle small sized sample volumes holds the future for biomarker research in paediatric populations.

*Wilco de Jager* *Wilco de Jager* *Dirk Holzinger* *Dirk Holzinger* *Ger T. Rijkers* *Ger T. Rijkers*
